# Sex-Related Differences in Cardiovascular Risk in Adolescents with Overweight or Obesity

**DOI:** 10.31083/j.rcm2510353

**Published:** 2024-09-29

**Authors:** Procolo Di Bonito, Anna Di Sessa, Maria Rosaria Licenziati, Domenico Corica, Malgorzata Wasniewska, Emanuele Miraglia del Giudice, Anita Morandi, Claudio Maffeis, Maria Felicia Faienza, Enza Mozzillo, Valeria Calcaterra, Francesca Franco, Giulio Maltoni, Nicola Moio, Arcangelo Iannuzzi, Giuliana Valerio

**Affiliations:** ^1^Department of Internal Medicine, “S. Maria delle Grazie” Hospital, 80078 Pozzuoli, Italy; ^2^Department of Woman, Child and of General and Specialized Surgery, Università degli Studi della Campania “Luigi Vanvitelli”, 80138 Napoli, Italy; ^3^Neuro-Endocrine Diseases and Obesity Unit, Department of Neurosciences, Santobono-Pausilipon Children's Hospital, 80129 Naples, Italy; ^4^Department of Human Pathology in Adulthood and Childhood, University of Messina, 98122 Messina, Italy; ^5^Department of Surgery, Dentistry, Pediatrics and Gynecology, Section of Pediatric Diabetes and Metabolism, University and Azienda Ospedaliera Universitaria Integrata of Verona, 37126 Verona, Italy; ^6^Pediatric Unit, Department of Precision and Regenerative Medicine and Ionian Area, University of Bari “Aldo Moro”, 70124 Bari, Italy; ^7^Department of Translational Medical Science, Section of Pediatrics, Regional Center of Pediatric Diabetes, University of Naples “Federico II”, 80131 Napoli, Italy; ^8^Pediatric Department, “V. Buzzi” Children's Hospital, 20154 Milano and Department of Internal Medicine, University of Pavia, 27100 Pavia, Italy; ^9^Pediatric Department, Azienda Sanitaria Universitaria del Friuli Centrale, Hospital of Udine, 33100 Udine, Italy; ^10^Pediatric Unit, IRCCS Azienda Ospedaliero-Universitaria di Bologna, 40138 Bologna, Italy; ^11^Department of Cardiology, “S. Maria delle Grazie” Hospital, 80078 Pozzuoli, Italy; ^12^Department of Medicine and Medical Specialties, A. Cardarelli Hospital, 80131 Naples, Italy; ^13^Department of Medical, Movement and Wellbeing Sciences, University of Napoli “Parthenope”, 80133 Napoli, Italy

Dear Dr. Saleh,

Thank you for your letter to the Editor regarding our article titled “Sex-Related Differences in Cardiovascular Risk in Adolescents with Overweight or Obesity”. We appreciate your interest in our work and welcome your stimulating comments that allowed us to better specify the protocol of carotid intima-media thickness (cIMT) measurement performed in our study.

First, we reply to the question on whether a single-site measure of cIMT in the common carotid artery or a multiple-site assessment in different carotid sections (common carotid artery, bifurcation and internal carotid artery) should be performed. The choice to measure cIMT only in the common carotid artery is justified by the easier and much more accurate measurement on this segment compared to the bulb or the internal carotid artery. Ultrasonographic evidence of atherosclerotic plaques is frequent in adults with hypertension, diabetes or other cardiovascular risk factors, and in children with homozygous familial hypercholesterolemia; their presence worsen the cardiovascular risk. Differently from adults, carotid plaques are very uncommon in children with obesity or overweight and most of the authors who have published data on subclinical atherosclerosis in children limited the ultrasonographic exploration of the carotid tree to the common carotid artery [1, 2, 3, 4]. However, the standardized examination protocol used in the present study included an initial quick transverse scan to identify focal thickenings of common carotid artery, bifurcation, and internal carotid artery. None of the subjects participating in this study had any plaque at these sites.

Regarding the question on whether cIMT should be measured only in the far wall or also in the near wall, we believe that measuring only the far wall (sometimes with a single scan angle) saves time and resources [5]. We are aware that this choice may often lead to misclassification of subjects, who sometimes have greater thickness on the near wall than on the far wall. Therefore, to reduce variability, three scanning angles were used in this study in each participant: anterior oblique, lateral and posterior oblique. According to a previous study published by our group in hypercholesterolemic children using the same ultrasonographic methodology reported in the present study, the average coefficients of variation for mean thickness were quite similar: near wall, 2.9%; far wall, 3.0%; and both walls, 2.4% [6]. Indeed, these coefficients of variation were by far less than 6%, the threshold recommended by the Association for European Paediatric Cardiology Working Group on Cardiovascular Prevention [7]. Therefore, the risk that manual cIMT measurements might have contributed to inaccuracy in cIMT estimation is largely attenuated. About this question, we also quote Ravani *et al*. [5] who stated that “cIMT measurements performed directly with the electronic caliper of the ultrasonic device itself are accurate enough and allow to detect associations with vascular risk factors or, possibly, to perform individual cardiovascular (CV) risk stratification” [8].

Lastly, the influence of the cardiac cycle on cIMT measurement was also underscored in the letter. In our study, cIMT was measured in both systole and end-diastole, along with the carotid artery diameters. Values reported in our study refer to data in end-diastole.

Anyway, we recognize that some procedural techniques employed in our study, such as the use of manual measurements could represent a flaw in the accuracy in cIMT estimation. Therefore, in view of this debate, our results relative to cIMT need to be considered cautiously. However, despite the different procedures, our findings in adolescents with obesity are in agreement with previous research in normal pediatric populations, that presented higher cIMT in boys than in girls [9, 10]. In these studies, measurements were performed only on the far wall with a semi-automated edge detection through a computer software package, with coefficients of variation similar to that reported by our group [6].
